# Detection of Zika virus using reverse-transcription LAMP coupled with reverse dot blot analysis in saliva

**DOI:** 10.1371/journal.pone.0192398

**Published:** 2018-02-05

**Authors:** Maite Sabalza, Rubina Yasmin, Cheryl A. Barber, Talita Castro, Daniel Malamud, Beum Jun Kim, Hui Zhu, Richard A. Montagna, William R. Abrams

**Affiliations:** 1 Department of Basic Sciences, New York University College of Dentistry, New York, New York, United States of America; 2 Rheonix, Inc., Ithaca, New York, United States of America; 3 Stomatology Department, School of Dentistry, University of São Paulo, São Paulo, São Paulo, Brazil; 4 Department of Medicine, New York University School of Medicine, New York, New York, United States of America; University of Helsinki, FINLAND

## Abstract

In recent years, there have been increasing numbers of infectious disease outbreaks that spread rapidly to population centers resulting from global travel, population vulnerabilities, environmental factors, and ecological disasters such as floods and earthquakes. Some examples of the recent outbreaks are the Ebola epidemic in West Africa, Middle East respiratory syndrome coronavirus (MERS-Co) in the Middle East, and the Zika outbreak through the Americas. We have created a generic protocol for detection of pathogen RNA and/or DNA using loop-mediated isothermal amplification (LAMP) and reverse dot-blot for detection (RDB) and processed automatically in a microfluidic device. In particular, we describe how a microfluidic assay to detect HIV viral RNA was converted to detect Zika virus (ZIKV) RNA. We first optimized the RT-LAMP assay to detect ZIKV RNA using a benchtop isothermal amplification device. Then we implemented the assay in a microfluidic device that will allow analyzing 24 samples simultaneously and automatically from sample introduction to detection by RDB technique. Preliminary data using saliva samples spiked with ZIKV showed that our diagnostic system detects ZIKV RNA in saliva. These results will be validated in further experiments with well-characterized ZIKV human specimens of saliva. The described strategy and methodology to convert the HIV diagnostic assay and platform to a ZIKV RNA detection assay provides a model that can be readily utilized for detection of the next emerging or re-emerging infectious disease.

## Introduction

Multiple outbreaks of infectious diseases, including yellow fever, severe acute respiratory syndrome (SARS), Ebola, dengue, chikungunya, are occurring with increasing frequency. At least three major factors have influenced the increasing incidence of infectious disease epidemics: population concentration at the epicenter of an infection, the underlying characteristics and vulnerabilities of the population at the epicenter [1] and extensive air travel to spread the disease globally [2–5]. The most recent is Zika virus (ZIKV), which is an emerging arbovirus in the genus *Flavivirus* and family of *Flaviviridae*, related to yellow fever, dengue, West Nile, Japanese encephalitis, and tick-borne encephalitis viruses. Zika virus is transmitted among humans primarily by mosquito vectors (*Aedes* spp., including *Ae*. *aegypti* and *Ae*. *Albopictus*) [6–8] and humans serve as the primary amplification hosts in areas where there are no non-human primates [9]. ZIKV was first identified in Uganda in 1947, and since then outbreaks have been recorded in Africa, Southeast Asia, Pacific with the last one in Brazil in early 2015, which spread to other countries in Central and South America, the Caribbean, and the Southern United States [10–12]. In all these areas autochthonous and travel associated infection has been reported [6–8, 13].

Early pathogen identification is necessary to combat the spread of these newly emerging and re-emerging epidemics [14, 15]. Diagnosis can be accomplished with a generic platform and protocol designed to identify any pathogen by detection of the pathogen’s nucleic acid once its sequence is determined and its antigenic footprint identified. The same platform can be utilized to detect any emerging or reemerging pathogen by modifying the primers and probes to detect specific target nucleic acid sequences and once identified, antigenic epitopes that can be utilized to detect pathogen specific antibodies.

In this study we have adapted a portable microfluidic device that we developed for HIV RNA detection to detect ZIKV RNA in saliva samples. We previously reported the ability to utilize a single sample of blood and/or saliva to simultaneously detect both HIV antibodies and HIV RNA using a microfluidic device for Point-of-Care (POC) testing [16]. We optimized a commercially available serological assay to run within the microfluidic device while we incorporated the reverse-transcription loop-mediated isothermal amplification (RT-LAMP) assay to detect the presence of HIV RNA.

LAMP was first reported by Notomi et al. 2000 [17] (Eiken Chemical Co.) and has rapidly gained popularity for POC testing [18, 19]. This is in part due to the relative simplicity of building an isothermal device, but also due to the strand displacement quality of the polymerase so there is no need to denature double stranded DNA prior to amplification. LAMP uses two separate sets of primer pairs that specifically recognize 6 distinct target regions. Addition of a third pair of primers (forward and reverse loop primers) increases the sensitivity and decreases the time required for results [20]. RNA can also be targeted with the addition of reverse transcriptase.

Saliva is a good choice for diagnostics due to the noninvasive ease of collection, cost effectiveness, and high acceptance by subjects/patients [21]. Saliva has been used for the diagnosis of salivary gland tumors [22], tongue squamous cell carcinoma [23], measurement of cortisol levels [24], viruses [25], RNA [26], and drugs of abuse [27].

In this study we first optimized the RT-LAMP assay to detect ZIKV RNA using a benchtop isothermal amplification device and then adapted the assay to the microfluidic device, Rheonix CARD^®^ cartridge. We have demonstrated that a fully integrated RT-LAMP assay coupled to reverse dot-blot (RDB) technique and processed by the Encompass *Optimum* workstation [28] can detect ZIKV RNA spiked in saliva samples. Having established a proof-of-concept of our methodology future experiments will be focused on validating it with human clinical samples.

## Materials and methods

### Saliva samples

Whole Mouth Stimulated Saliva (WMSS) was selected for use in this study because it is less viscous than other oral fluids. WMSS samples were collected as previously described by Chen et al. 2016 [16] under the protocol (H10-01894) approved by the Institutional Review Broad of the New York University Langone School of Medicine.

### Zika viral production and titration

ZIKV strain PRVABC59, NR-50240 was obtained through Biodefense and Emerging Infections Research Resources Repository (BEI Resources), NIAID, NIH and propagated in Vero cells. ZIKV stock was obtained from infected cells supernatant.

Vero E6 cells (ATCC^®^ CRL-1586TM) were maintained in tissue culture flasks at 37°C in an atmosphere containing 5% CO_2_ with Eagle’s minimum essential medium containing Earle’s balanced salt solution, non-essential amino acids, 2 mM L-glutamine and 1 mM sodium pyruvate, 100 units Penicillin, 0.1 mg/ml Streptomycin, 1.25% Amphotericin, supplemented with 2% fetal bovine serum (Atlanta Biologicals, S11150H).

Vero cells were infected when at 70% to 80% confluence at a multiplicity of infection (MOI) of 0.01. After 1 h virus adsorption at 37°C in an atmosphere containing 5% CO_2_, fresh medium was added and cells incubated for up to 3 days. The supernatant was then collected, centrifuged at 2000 x g for 5 min to remove cell debris, and the supernatant stored at -80°C. Viral titration was determined by plaque assay in Vero cells with a 0.4% ultrapure low melt point agarose (Invitrogen, 16520–050) overlay. After 5 days of incubation at 37°C in an atmosphere containing 5% CO_2_, cells were fixed with 10% formaldehyde, washed, and stained with 0.025% (wt/vol) crystal violet in 2% (vol/vol) ethanol for visualization of plaques (2.9 x 10^6^ plaque-forming units/ml). Genomic purified ZIKV RNA (PRVABC59, Puerto Rican strain, NR-50244) obtained from BEI Resources, NIAID, NIH (Manassas, VA) with a known viral RNA copy number was serially diluted ten-fold and used as a standard to generate a standard concentration curve graphing the RNA concentration (RNA copies/ml) vs amplification time in the RT-LAMP assay on the Genie III device. This allowed estimating the copy number of the ZIKV stock (2.2 x 10^7^ RNA copies /ml).

### Purified genomic RNA from ZIKV

Purified genomic RNA from ZIKV, strain PLCal_ZV (Thailand) (70 ng/μl, 1.6 x 10^13^ RNA copies/ml), was donated by Dr. James Whitney, Center for Virology and Vaccine Research, Beth Israel Deaconess Medical Center, Harvard Medical School. Additional purified genomic RNA from four different ZIKV strains (Asian lineage) were obtained through BEI Resources, (Manassas, VA): PRVABC59, Puerto Rican strain, NR-50244 (2.0 x 10^7^ RNA copies/ml); PLCal_ZV, Thailand strain, NR-50242 (5.1 x 10^7^ RNA copies/ml); FLR, Florida strain, NR-5024 (4.4 x 10^7^ RNA copies/ml) and R103451, Honduras strain, NR-50358 (5.4 x 10^7^ RNA copies/ml). The BEI Resources (Manassas, VA) supplied the copy numbers of viral RNA per ml (RNA copies/ml), determined using Droplet Digital RT-PCR.

Purified genomic RNA from Dengue Virus (DENGV) serotype 1 (Hawaii, NR-4287) and serotype 2 (New Guinea C, NR-4288) were also obtained through BEI Resources (Manassas, VA).

### LAMP primers

Primers targeting a unique conserved sequence in the ZIKV capsid were designed using the Primer Explorer V4 software (Eiken Chemical Co., Tokyo, Japan) with conserved regions of the capsid gene of the PLCal_ZV, Thailand strain (GeneBank accession no. KF993678.1). A set of 3 primer pairs (Table 1), including two outer primers (forward primer F3 and backward primer B3), two inner primers (forward inner primer FIP and backward inner primer BIP), and two loop primers (forward loop primer LF and backward loop primer LB), were selected. All primers were assessed for specificity before use in LAMP assays by analysis using the Basic Local Alignment Search Tool (BLAST) of the National Center for Biotechnology Information (NCBI) against sequences in GenBank indicating that they are ZIKV specific. The ZIKV primers were also tested in duplicate for cross-reactivity against Dengue virus (DENV) RNA, serotype 1 and 2. DENV RNA from both serotypes did not amplify with ZIKV primers, showing specificity of the ZIKV primers (S1 and S2 Figs). Sequence of the Dengue primers for each serotype used were described in Lau et al. 2015 [29].

**Table 1:** LAMP ZIKV primers and optimized concentrations.

Primer	5’3’ nucleotide sequence	Optimized concentration (μM)
F3	GACTTCTGCTGGGTCATG	0.4
B3	GCCAACAATTCCGACACTA	0.4
FIP*	CCCCACTGAACCCCATCTATTGGGTCTTGGCGATTCTAGC	2.4
BIP	GTTCAAGAAAGATCTGGCTGCCCCTCGTCTCTTCTTCTCCT	2.4
Loop F	GCTTGATTGCCGTGAATCTC	1.0
Loop B	GCTGAGAATAATCAATGCCAGG	1.0

* Biotinylated primer for RDB assay on the Rheonix CARD^®^ cartridge. μM: optimized primer concentration.

### RT-LAMP assay

RT-LAMP reaction was carried out in a final volume of 25 μl including the OptiGene Master mix ISO-004 with 7.5 units of WarmStart reverse transcriptase (New England BioLabs, M0380L) and 3 pairs of primers targeting a unique conserved sequence in the ZIKV capsid (Table 1). 3–7 μl RNA were added as a template. The RT-LAMP reaction was performed at 65°C for 30 min. The temperature and primer concentrations were optimized in the lab.

#### Real-time RT-LAMP assay using the Genie III device

Experiments using the Genie III (OptiGene, Horsham, UK) portable isothermal amplification device were performed to optimize the RT-LAMP assay. SYBR Green I interchelating dye is included in the OptiGene Master mix (OptiGene ISO-004) which allows following real-time fluorescence in a Genie III.

#### RT-LAMP assay using the microfluidic Rheonix CARD^®^ cartridge

The microfluidic Rheonix CARD^®^ cartridge developed by Rheonix Inc. (Ithaca, NY) is an integrated cassette that can provide both an immunological and nucleic acid (RT-LAMP) result from a single sample. It incorporates all the pumps, valves, micro channels, reactions, and reagent reservoirs on a disposable cartridge described in Chen et al. 2016 [16]. The processing and analysis on the Rheonix CARD^®^ cartridge is controlled by the Encompass *Optimum* workstation developed to provide automated control of all sample preparation functions as described in Spizz et al. 2012 [28]. Since the current Encompass *Optimum* workstation does not possess the ability to monitor fluorescence, we employed a novel reverse dot-blot (RDB) hybridization technique to monitor the specific ZIKV amplicons produced in the RT-LAMP assay using hybridization to nucleic acid probes immobilized on integrated DNA arrays [30]. Coupled with the workstation’s image analysis software, qualitative viral loads can be determined. In this approach 50 μl of sample containing pre-heated lysed ZIKV was added to the sample reservoir on the Rheonix CARD^®^ [16]. All other steps were automatically performed under software control. The RT-LAMP reaction mix was dispensed automatically into the master mix reservoir onto the Rheonix CARD^®^ cartridge [16] and pumped with 7 μl of the spiked sample to the amplification tube where the RT-LAMP reaction was carried out at 65°C for 30 min. ZIKV RNA was amplified using biotinylated primers. The RDB method employs the same primers used for the RT-LAMP assay on the Genie III (Table 1) but the FIP primer is biotinylated. After amplification, the denatured amplicons are hybridized to membrane bound ZIKV capture probes and detected by subsequent addition of streptavidin-conjugated HRP and substrate. Initially four DNA oligo probes were designed to target the ZIKV amplicon from the RT-LAMP reaction (Table 2). These four DNA probes were synthesized and immobilized on an activated membrane as a DNA array [28, 30] at three different concentrations (20, 2 and 0.2 μM). The microarray key is provided as supporting information (S1 Table). Pre-heated lysed ZIKV diluted in 1XPBS (phosphate buffered saline, pH 7.4) was used as a template for RDB assay optimization on the Rheonix CARD^®^ cartridge.

**Table 2:** LAMP ZIKV probes used in RDB assay.

ZIKV Probe	5’3’ nucleotide sequence
Probe 1	CACGGCAATCAAGCCATCACTGGGTC
Probe 2	TGGGGTTCAGTGGGGAAAAAAG
Probe 3	GGGGAAAAAAGAGGCTATGG
Probe 4	GGCTGCCATGCTGAGAATAATCAATGCC

In subsequent experiments, Probe 4 was immobilized on an activated membrane as a DNA array [28, 30] at different concentrations from 20 μM to 0.039 μM by two-fold dilutions. The microarray key is provided as supporting information (S2 Table). Pre-heated lysed ZIKV diluted in 1XPBS was used as a template for optimization of the RDB assay to semi-quantitative estimate the viral load. Pre-heated lysed ZIKV spiked in saliva was used as a template in the fully integrated LAMP assay plus RDB detection on the Rheonix CARD^®^ cartridge.

### Devices used for RT-LAMP assays

Depending on the device used for the RT-LAMP assay, different detection techniques were applied (Table 3).

**Table 3:** Devices used for RT-LAMP assay to detect ZIKV.

Device	RT-LAMP detection technique	Samples analyzed	Experiment
Genie III (Optigene, Hosham, UK)	Fluorescence	Purified genomic RNA; ZIKV spiked saliva	RT-LAMP assay optimization
Rheonix CARD^®^ cartridge controlled by the automated Encompass *Optimum* workstation	Reverse dot-blot	Pre-heated lysed ZIKV spiked in 1XPBS	RDB optimization
		Pre-heated lysed ZIKV spiked saliva	Full-integrated RT-LAMP and RDB assays

### Spiking saliva samples

Uninfected human saliva samples were spiked with ZIKV to test the feasibility of using a RT-LAMP assay with saliva samples. Ten-fold serial dilutions of the virus in 1XPBS were prepared. Saliva was first diluted 1:5 and 10 μl of each ten-fold serial dilution of the virus was spiked into saliva and then heated at 90°C for 5 min to inactivate and lyse the virus. Inactivation of ZIKV under these conditions was confirmed with plaque assays using Vero cells as described above (S1 Table and S3 Fig). For experiments performed on the Rheonix CARD^®^ cartridge the uninfected human saliva was spiked as above but with pre-heated inactivated ZIKV. This safety measure was required because the facilities at Rheonix Inc. (Ithaca, NY) laboratory are not at a BSL-2 level.

## Results

### ZIKV RNA RT-LAMP assay limit of detection on the Genie III device

**Fig 1:**
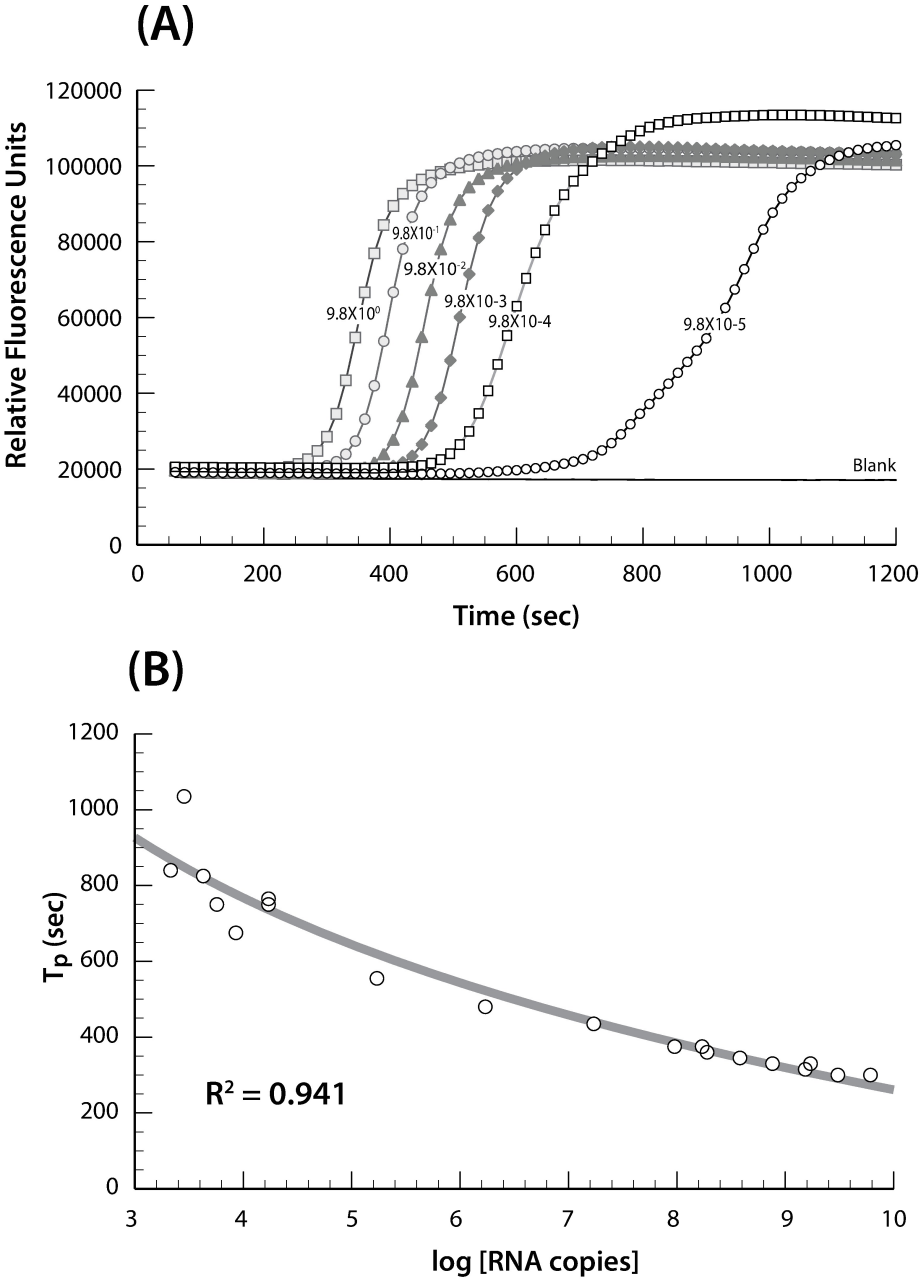
ZIKV RNA RT-LAMP amplification. (A) Representative RT-LAMP amplification curves that target the ZIKV capsid gene. Ten-fold serial dilutions of purified genomic RNA starting at 10 ng/ul. LAMP assay performed on the Genie III device. (B) Graph of the amplification threshold Tp as a function of ZIKV viral particle concentration. Tp: time to positivity.

Using the Genie III device we first demonstrated that a RT-LAMP assay could be used for the detection of ZIKV RNA by testing serial ten-fold dilutions in 1XPBS of purified ZIKV genomic RNA (strain PLCal_ZV (Thailand) donated by Dr. Whitney) (Fig 1A). Results showed that the amplification time values, also known as Tp (time to positivity), of the RT-LAMP for ten-fold serial dilutions of ZIKV RNA detection were between 3 and 10 min (Fig 1A) and a linear relationship between ZIKV RNA concentration and signal intensity was observed. The linear relationship of the RT-LAMP assay can be seen by the relationship between the log viral RNA copies per ml against the Tp values (Fig 1B).

We also demonstrated that the RT-LAMP assay using primers targeting the ZIKV capsid gene could detect multiple ZIKV Asian-lineages strains. For these experiments all the strains concentrations were adjusted to 2 x 10^6^ RNA copies/ml. We determined the limit of detection of the RT-LAMP assay using ten-fold serial dilutions of purified genomic RNA from 4 different ZIKV strains of the Asian-lineage (obtained from BEI, NIAID, NIH). The results indicate that the RT-LAMP assay using primers that target the capsid consistently detected down to 2 x 10^3^ (6 RNA copies/reaction) (Table 4).

**Table 4:** Limit of detection of RT-LAMP assay for ZIKV RNA from Asian-lineages ZIKV strains.

	RT-LAMP assay positive sample, n = 3			
ZIKV RNA copies/ml	PRVABC59 (Puerto Rico)	PLCal_ZV (Thailand)	FL (Florida)	R103451 (Honduras)
2.0 x 10^7^	3/3	3/3	3/3	3/3
2.0 x 10^6^	3/3	3/3	3/3	3/3
2.0 x 10^5^	3/3	3/3	3/3	3/3
2.0 x 10^3^	3/3	3/3	3/3	3/3
2.0 x 10^2^	1/3	1/3	2/3	1/3
2.0 x 10^1^	0/3	0/3	0/3	0/3

### ZIKV RNA is detectable in saliva

Uninfected human saliva was spiked with ZIKV at different concentrations corresponding to ten-fold serial dilutions. RT-LAMP assay was performed on the Genie III device. Consistently, the RT-LAMP assay detected 2.2 x 10^3^ RNA copies/ml (6.6 RNA copies/reaction) in 100% of the samples and in 75% of the samples 2.2 x 10^2^ RNA copies/ml (0.66 RNA copies//reaction) (Table 5). Ten replicates per dilution were tested.

**Table 5:** Limit of detection of RT-LAMP assay using spiked saliva samples with ten-fold serial dilutions of ZIKV.

Spiked ZIKV in saliva (PRVABC59) (n = 10)		RT-LAMP assay	
ZIKV Concentration (RNA copies/ml)	LAMP Reaction (RNA copies per reaction)	Tp (min) Mean (95%CI)	Positive samples in 10 replicates
2.2 x 10^5^	660.00	8.36 (7.5, 9.15)	10/10
2.2 x 10^4^	66.00	9.43 (8.56, 10.29)	10/10
2.2 x 10^3^	6.60	11.45 (8.44, 14.46)	10/10
2.2 x 10^2^	0.66	19.95 (2.14, 37.76)	7/10
2.2 x 10^1^	0.06	ND	ND
0	0.00	ND	ND

Tp: time to positivity; ND: no fluorescence signal detected; RT-LAMP assay performed on the Genie III device. Tp of ten technical replicates. Values are represented as the mean and 95% Confident interval (CI).

### Microfluidic Rheonix CARD^®^ cartridge adapted to detect ZIKV RNA

**Fig 2:**
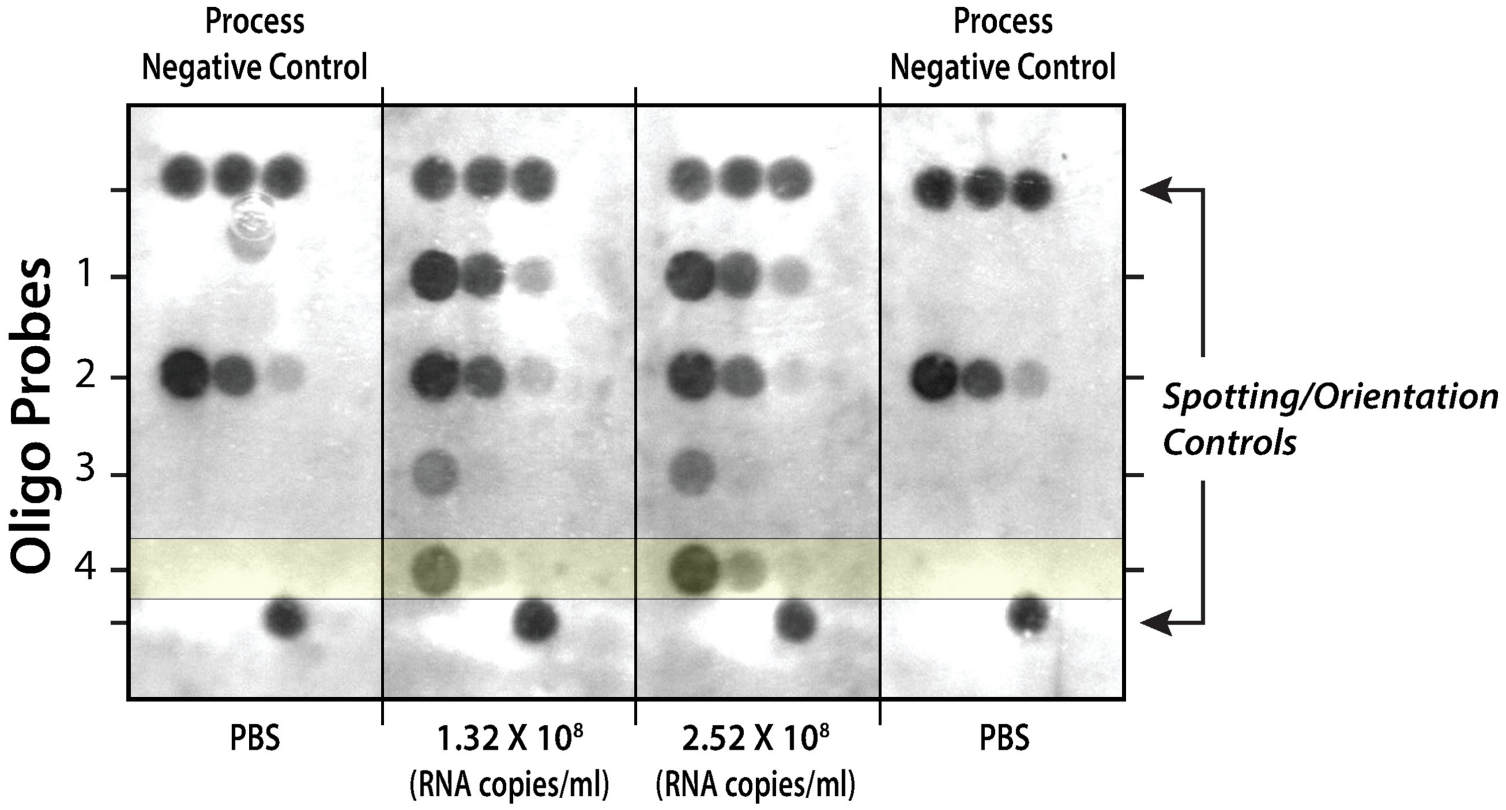
Detection of RT-LAMP amplicons on DNA array under the control of the Encompass *Optimum* workstation. Four probes at three different concentrations (20, 2 and 0.2 μM) were designed to react with either the primer sets (Probes 1 or 2) or the RT-LAMP amplicons (Probes 3 or 4). The “Spotting Controls” should be positive in all reactions and allow the system’s software to confirm that the DNA arrays are properly orientated as well as provide a reference for alignment of the CMOS camera when analyzing the spot intensities. Process Negative Control: 1XPBS used as a template for RT-LAMP assay. Pre-heated lysed ZIKV diluted in 1XPBS was used as template.

Pre-heated lysed ZIKV diluted in 1XPBS as template at 2 different concentrations (1.32 x 10^8^ and 2.52 x 10^8^ RNA copies/ml) was used to optimize the experiments on the Rheonix CARD^®^ cartridge. The RT-LAMP assay with biotinylated labeled primers was automatically carried out in the amplification tube in the Rheonix CARD^®^ cartridge. Representative results show (Fig 2) that the RT-LAMP amplicons can be detected using the RDB technique in the Rheonix CARD^®^ cartridge. These results also showed that Probes 1 and 2 hybridized with the primers for the RT-LAMP reaction and thus were not specific. However, Probes 3 and 4 are specific for ZIKV amplicon (Fig 2) with Probe 4 (highlighted in yellow) generating the strongest signal. The microarray key is provided as supporting information (S2 Table).

**Fig 3:**
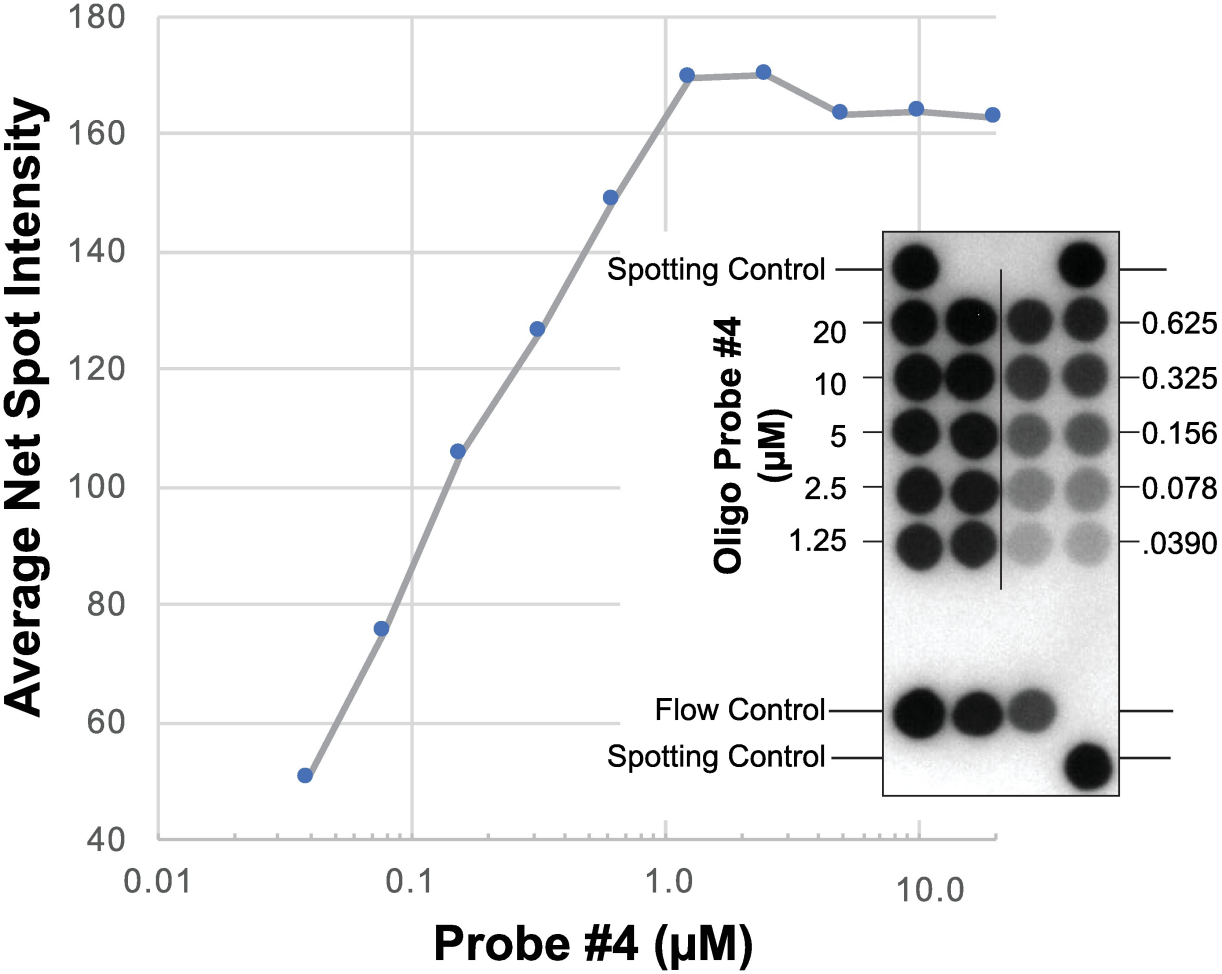
Dose response graph of Probe 4 hybridization intensity as a function of its micro molar concentration at a fixed level of ZIKV amplicon. The insert is the image of the hybridization plot used to generate the data. The image is annotated to show the spotting and flow controls. The “Spotting Controls” should be positive in all reactions and allow the system’s software to confirm that the DNA arrays are properly orientated as well as providing a reference for alignment of the CMOS camera when analyzing the spot intensities. RT-LAMP assay performed on the Rheonix CARD^®^ cartridge under the control of the Encompass *Optimum* workstation. Pre-heated lysed ZIKV diluted in 1XPBS was used as template.

Probe 4 was selected for further RDB experiments. A serial dilution of Probe 4, which binds specifically to ZIKV amplicons, demonstrates that it is possible to semi-quantitatively determine the viral load based on the image density of the hybridized spot (Fig 3). Probe 2, which binds to the primer pairs was also included on the array to confirm that the reaction mix containing the ZIKV primer pairs and amplicon was successfully pumped from the Rheonix CARD^®^ cartridge’s reservoirs to the DNA array.

**Fig 4:**
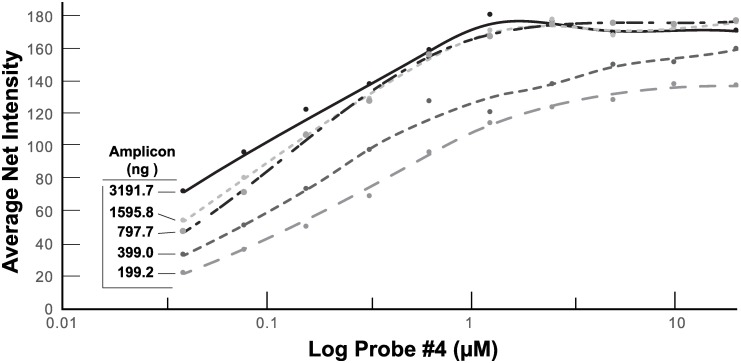
Titration of ZIKV RT-LAMP amplicons against concentrations of Probe 4. The amplicon concentration was determined by Nanodrop and serially diluted in 1XTE (10mM Tris +1mM EDTA). 5 μl of each dilution of known concentrations were used to hybridize the filters spotted with concentrations of Probe 4. The intensity of each spot with known concentrations of amplicon was analyzed by Image J software and plotted. Pre-heated lysed ZIKV diluted in 1XPBS was used as template.

To further demonstrate the ability to obtain a semi-quantitative estimation of the viral load, we evaluated the relative signal intensity of a range of ZIKV RT-LAMP amplicons (~199 ng to 3200 ng) as a function of increasing concentration of the biotinylated Probe 4 (Fig 4) and an excellent dose response curve was achieved. An increase of sensitivity was observed with high concentrations of amplicon and Probe 4. Depending on the amplicon concentration generated by LAMP, the response reaches maximum intensity as a function of immobilized Probe 4 concentration. Future experiments will focus on evaluating Probe 4 concentration to obtain a linear dose response allowing a more accurate estimation of the viral load of the sample analyzed.

**Fig 5:**
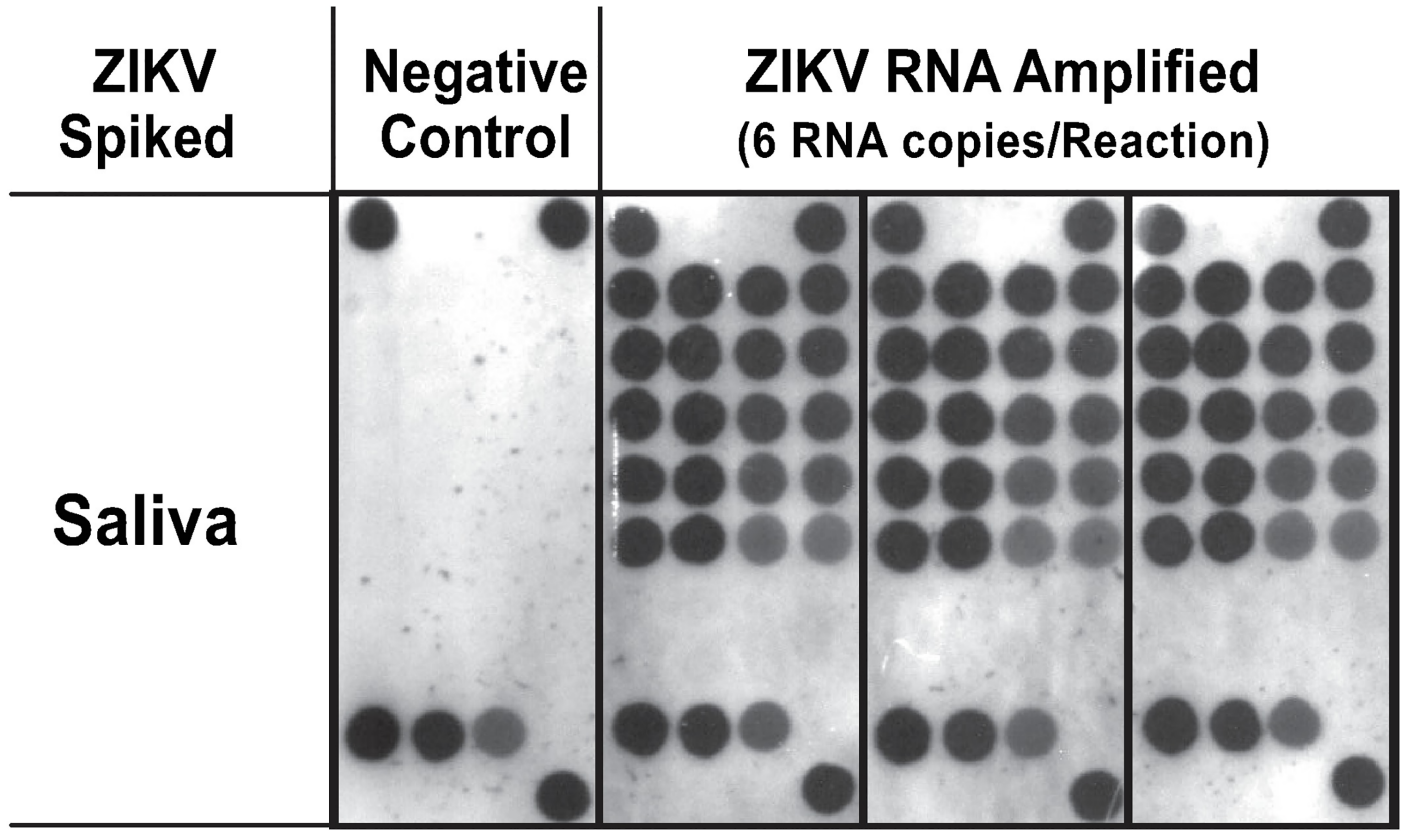
Fully integrated RT-LAMP assay, performed on the microfluidic Rheonix CARD^®^ cartridge under the control of the Encompass *Optimum* workstation in which the presence of the amplicons was detected on the integrated DNA array. Each spot on the array represents duplicate immobilized Probe 4 ranging from a concentration of 20 μM to 0.039 μM by two-fold dilutions as described in Fig 3. A total of 3 replicates of saliva spiked with 6 ZIKV RNA copies per reaction were evaluated along with negative controls (1XPBS as a template).

In order to determine the ability to perform a fully integrated LAMP assay including RDB detection on the Rheonix CARD^®^ cartridge, we performed an assay using pre-heated lysed ZIKV spiked in saliva samples as a template. The saliva samples containing pre-heat lysed ZIKV were dispensed into the sample reservoir of the Rheonix CARD^®^ cartridge and then pumped into each amplification tube along with LAMP master mix for RT-LAMP reaction followed by RDB processing for visualization of the reaction product amplicons. The results indicate detection in saliva samples down to 6 ZIKV RNA copies (Fig 5). The microarray key is provided as supporting information (S3 Table).

## Discussion

The recent ZIKV outbreak is an example of the course of emerging infectious diseases. ZIKV infection had been considered to only be a modest public health concern with only local outbreaks [31, 32]. However reports from the recent outbreak in Brazil and the Americas during 2015–2016, indicated that infections during pregnancy are associated with microcephaly and other neurological disorders in newborns and Guillain-Barré Syndrome (GBS) in adults pointing to potentially more serious health impacts [33, 34]. On 1 February 2016, the World Health organization (WHO) declared ZIKV and its link to birth defects a public health emergency of international concern and it lasted during 10 months [35]. ZIKV diagnosis is a challenge because it shares vectors, geographic distribution and symptoms with Dengue virus and Chikungunya infection [36] and the three illnesses are often misdiagnosed. Given the risk for adverse pregnancy outcomes in women infected with ZIKV during pregnancy, it is particularly important to distinguish between the 3 viruses. This recent ZIKV outbreak confirm that we need an effective surveillance and diagnostic program to reduce the impact of future emerging infectious diseases. Towards the goal of increasing the speed of making effective diagnostics available, we have developed a generic protocol that can be adapted for the next emerging or remerging disease.

Although PCR is a sensitive assay used for diagnostics, PCR requires trained personnel, dedicated equipment and extended time (hours) for results. Isothermal amplification methods have increasingly attracted attention due to their simplicity and sensitivity including nucleic acid sequence-based amplification (NASBA), loop-mediated isothermal amplification (LAMP), helicase-dependent amplification (HDA), rolling circle amplification (RCA), multiple displacement amplification (MDA) and recombinase polymerase amplification (RPA) [37–40] Among commercially available isothermal systems, LAMP stands out for its rapidity, sensitivity and ability to amplify both DNA and RNA [17]. This is why we chose the LAMP assay to detect HIV viral RNA in the HIV diagnostic dual assay that we developed [16]. Then we successfully adapted it to detect DNA from *P*. *falciparum* and *P*. *vivax* by simply changing the primers and optimizing the LAMP assay [41].

Salivary testing for diagnosis has advantages over other bodily fluids [21, 42] but in particular in ZIKV diagnosis it is beneficial because ZIKV RNA can only be detected in the blood from 1–7 days after the clinical onset during acute infection, but persists longer in saliva, semen and urine [43–45] suggesting that saliva could be a preferred specimen to use for ZIKV diagnostics. Drawbacks to saliva as a test matrix are that the concentration is frequently lower than typically found in serum and circadian variations in analyte concentrations can make the sensitivity lower [46]. However recent development of microfluidic concentrators suitable for diagnostics makes the analysis of nucleic acid targets that are present in low levels [47] possible.

We first optimized the RT-LAMP assay using ZIKV purified genomic RNA as a template on the Genie III device. We focused on the Asian-lineage ZIKV strains because they were responsible for the recent epidemics in Latin America. There is a significant variation in sequence between the African and Asian-lineages [48, 49]. Therefore other primers will have to be designed to specifically detect the African lineage. We also determined the limit of detection of the assay with a dilution series of purified genomic ZIKV RNA from multiple strains of the Asian-lineage. ZIKV RNA levels of 2 x 10^3^ RNA copies/ml (6 RNA copies/reaction) were consistently detected in the four strains. The limit of detection of the RT-LAMP assay using spiked saliva samples was also found to be ~2 x 10^3^ RNA copies/ml (6.6 RNA copies/reaction).

There have been reports showing that ZIKV can be detected by RT-LAMP assay without the need for RNA purification [50, 51]. Our experiments also demonstrated that heat-lysis of ZIKV is sufficient and subsequent isolation and purification of viral RNA is not required. In spiking experiments, saliva samples were diluted 1:5 before isothermal amplification in order to avoid the influence of matrix during real-time fluorescence detection. The heat-lyse treatment protocol is easier, faster and requires less sample volume for the LAMP assay than isolation and purification, which increases the compatible with POC applications.

After showing that the RT-LAMP assay on the Genie III could detect purified genomic RNA as well as ZIKV spiked in saliva samples, we adapted it to the microfluidic Rheonix CARD^®^ cartridge and processed it by the Encompass *Optimum* workstation. This approach successfully detects ZIKV RNA using the RDB technology on the Rheonix CARD^®^ cartridge and coupling this with the workstation’s image analysis and software capability provides a semi-quantitative titer of viral load based on the image density of the hybridized spot. Estimating the viral load can be useful to investigate correlations of viral load with disease severity and immune responses. This would be of particular importance for monitoring fetal infections in pregnant women [52, 53]. We demonstrated the ability to perform a fully integrated RT-LAMP assay including RDB detection on the Rheonix CARD^®^ cartridge using ZIKV spiked in saliva down to 8.57 x 10^2^ RNA copies/ml (6 RNA copies/reaction). The same limit of detection was obtained for the RT-LAMP assay on the Genie III with saliva samples spiked with ZIKV. Ongoing experiments are focused on optimizing the conditions for amplification of ZIKV RNA directly by heat-lysing the virus in the Rheonix CARD^®^ cartridge and without purification of ZIKV RNA. Eventually the entire assay will be validated on Rheonix CARD^®^ cartridges using well-characterized ZIKV human specimens as well as samples from people infected with other flaviviruses to confirm the reactivity and specificity of the assay.

The limit of detection of our RT-LAMP assay on the Genie III and Rheonix CARD^®^ cartridges is similar to other results reported using RT-LAMP and other isothermal assays [54]. These are promising results for the RT-LAMP assay coupled with RDB detection assay developed for the Rheonix CARD^®^ cartridge. However these results need to be validated using ZIKV samples from well-characterized human specimens.

In the HIV dual diagnostic assay that we previously developed, the Rheonix CARD^®^ cartridge was processed in the prototype Encompass MDx SOLO workstation providing viral RNA isolation and detection via RT-LAMP as well as antibody detection [16]. The compact and portable real-time Encompass MDx SOLO workstation can process a single Rheonix CARD^®^ cartridge, allowing a total of 4 samples to be simultaneously processed with real-time fluorescence monitoring. We moved to the larger Encompass *Optimum* Workstation because it allows 1 to 6 Rheonix CARD^®^ cartridges (24 samples) to be processed simultaneously. Since the Encompass *Optimum* workstation does not employ fluorescence excitation and detection system, the RDB technique was employed to interpret the molecular results by use of hybridization to nucleic acid probes immobilized on integrated DNA arrays. A major advantage of the Encompass *Optimum* workstation over the Encompass MDx SOLO workstation is that the *Optimum* workstation automatically introduces all samples and reagents to the Rheonix CARD^®^ cartridge, which reduces the complexity of the final assay for the end user. In addition user friendly and intuitive software is included in the workstation guiding the user. A touchscreen initiates the totally automated process and finally interprets and reports the assay results.

In ZIKV infections, the virus persists in plasma or serum for only a relatively short period of time while ZIKV can be detected for extended periods in other bodily fluids such as saliva, urine or semen [55, 56]. This supports the goal to combine serological assays and RNA assays to provide clinically relevant information across a longer time frame, which is particularly useful in treating pregnant women.

The ZIKV serological assay is challenging because ZIKV antigens cross-react with other flavivirus amino acid sequences [57]. We are working on the identification of ZIKV specific antigens using a high-density peptide microarray (PEPperCHIP Platform Technology, Heidelberg, Germany) whereby 15-mer amino acids, overlapping by 14 amino acids are probed with antibodies of interest in order to identify immunogenic epitopes [58]. Once validated the identified diagnostic peptides will be adapted to a serological assay incorporated on the Rheonix CARD^®^ cartridge using a modification of the approach we used for antibody detection against HIV [16]. Then we will able to simultaneously detect anti-ZIKV antibodies and RNA using the Rheonix CARD^®^ cartridges.

There are only a few studies that have reported ZIKV detection in saliva using RT- LAMP [51, 59, 60]. The novelty of our developed platform is the use of saliva as the sample matrix and the RDB detection system coupled to the RT-LAMP assay performed in the Rheonix CARD^®^ cartridges. In addition, once diagnostic peptides are validated and incorporated into the Rheonix CARD^®^ cartridges, we will be able to simultaneously detect ZIKV antibodies and ZIKV RNA. There is currently no combined antibody/nucleic acid test available commercially for ZIKV detection [61, 62].

Adaption of this protocol for the next emerging infection requires two steps: (1) identification of the nucleic acid sequence of the infectious agent, which allows synthesis of LAMP primers and associated RDB probes and (2) discovery of diagnostic peptides that detect host immune response(s) for a protein microarray. Our results provide the proof-of-principle of a protocol for adapting an existing LAMP assay on a microfluidic device to detect HIV RNA, to an assay to detect ZIKV RNA. Further experiments with clinical samples and diagnostic peptides will validate the assay.

## Supporting information

S1 Fig ZIKV RT-LAMP primers specificity. Graph of the amplification time (seconds) as a function of the fluorescence signal monitored with the Genie III device. No amplification detected in RT-LAMP assay using ZIKV capsid primers and DENGV serotype 1 (green) and 2 (purple) genomic purified RNA as templates. ZIKV genomic purified RNA was used as a template for positive control. Distilled water instead of RNA template was used as a negative control in the RT-LAMP assay.
(TIF)

S2 Fig DENGV RT-LAMP primers specificity. Graph of the amplification time (seconds) as a function of the fluorescence signal monitored with the Genie III device. No amplification detected in RT-LAMP assay using DENGV serotype 1 (blue) and 2 (red) genomic purified RNA as templates. DENGV serotype 1 (green) and 2 (purple) genomic purified RNA was used as a template for positive control. Distilled water instead of RNA was used as a negative control in the RT-LAMP assay.
(TIF)

S3 Fig Plaque assay on Vero cells infected with heat inactivated ZIKV. Infections at high and low MOIs with heat inactivated ZIKV at 90°C for 5 min did not show any detectable plaques. No heat inactivated ZIKV (no treatment) was used as a control for infected Vero cells. 2 replicates were used for high MOI infections and 3 for low MOI infection. The bottom well in the MOI 7 columns corresponds to Vero cells without virus used as an uninfected control.
(TIF)

S1 Table Plaque assay to test heat inactivated ZIKV. (DOCX)

S2 Table Microarray key for RDB assay. (DOCX)

S3 Table Microarray key for RDB assay using Probe 4. (DOCX)
