# Supplementary figures and images for: Detection of Zika virus using reverse-transcription LAMP coupled with reverse dot blot analysis in saliva
Source: PLoS One. 2018 Feb 5;13(2):e0192398. doi: 10.1371/journal.pone.0192398 (PMC5798782; doi:10.1371/journal.pone.0192398)

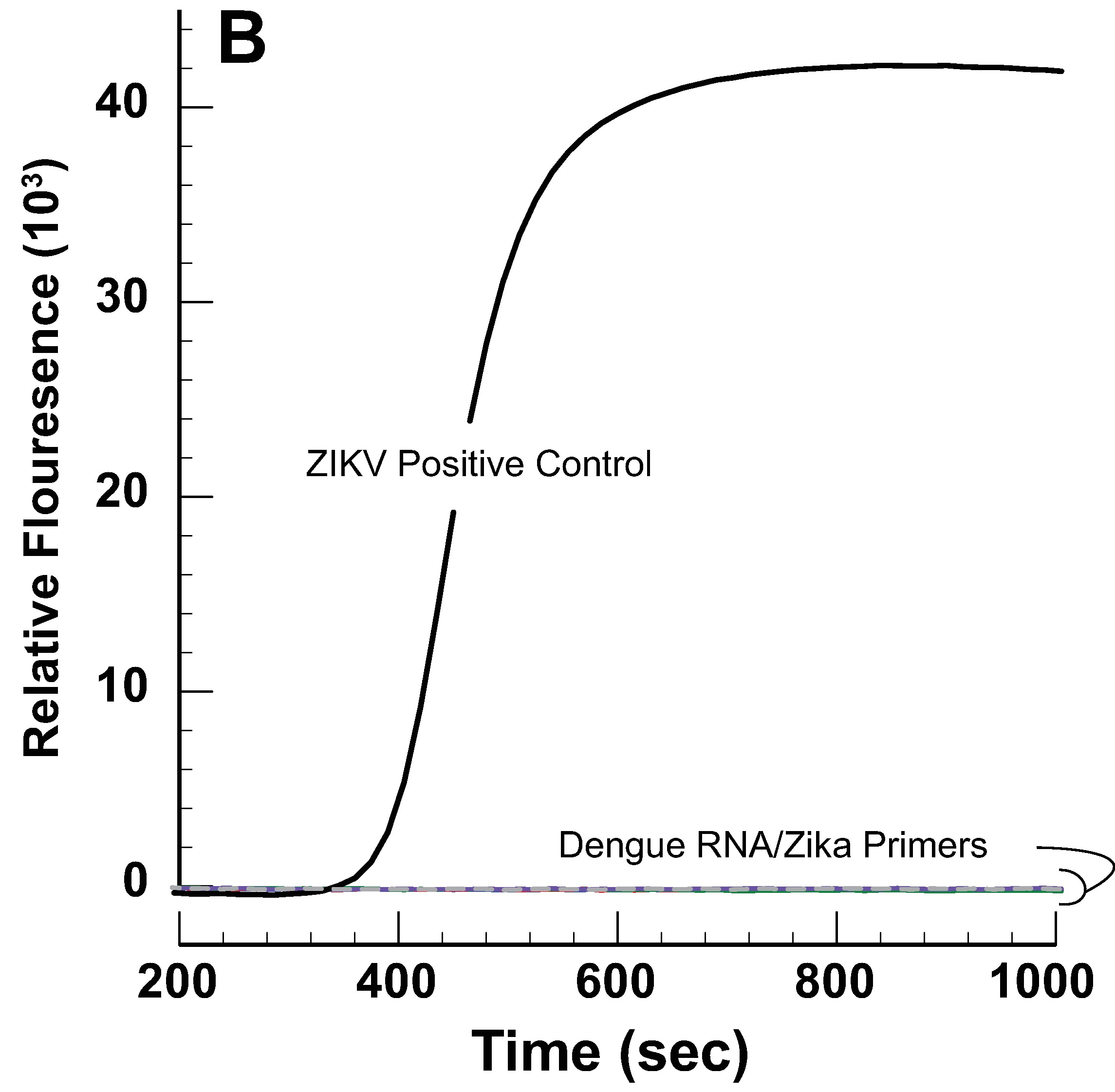

Supplement: S1 Fig — Graph of the amplification time (seconds) as a function of the fluorescence signal monitored with the Genie III device. No amplification detected in RT-LAMP assay using ZIKV capsid primers and DENGV serotype 1 (green) and 2 (purple) genomic purified RNA as templates. ZIKV genomic purified RNA was used as a template for positive control. Distilled water instead of RNA template was used as a negative control in the RT-LAMP assay. (TIF) [file pone.0192398.s001.tif]

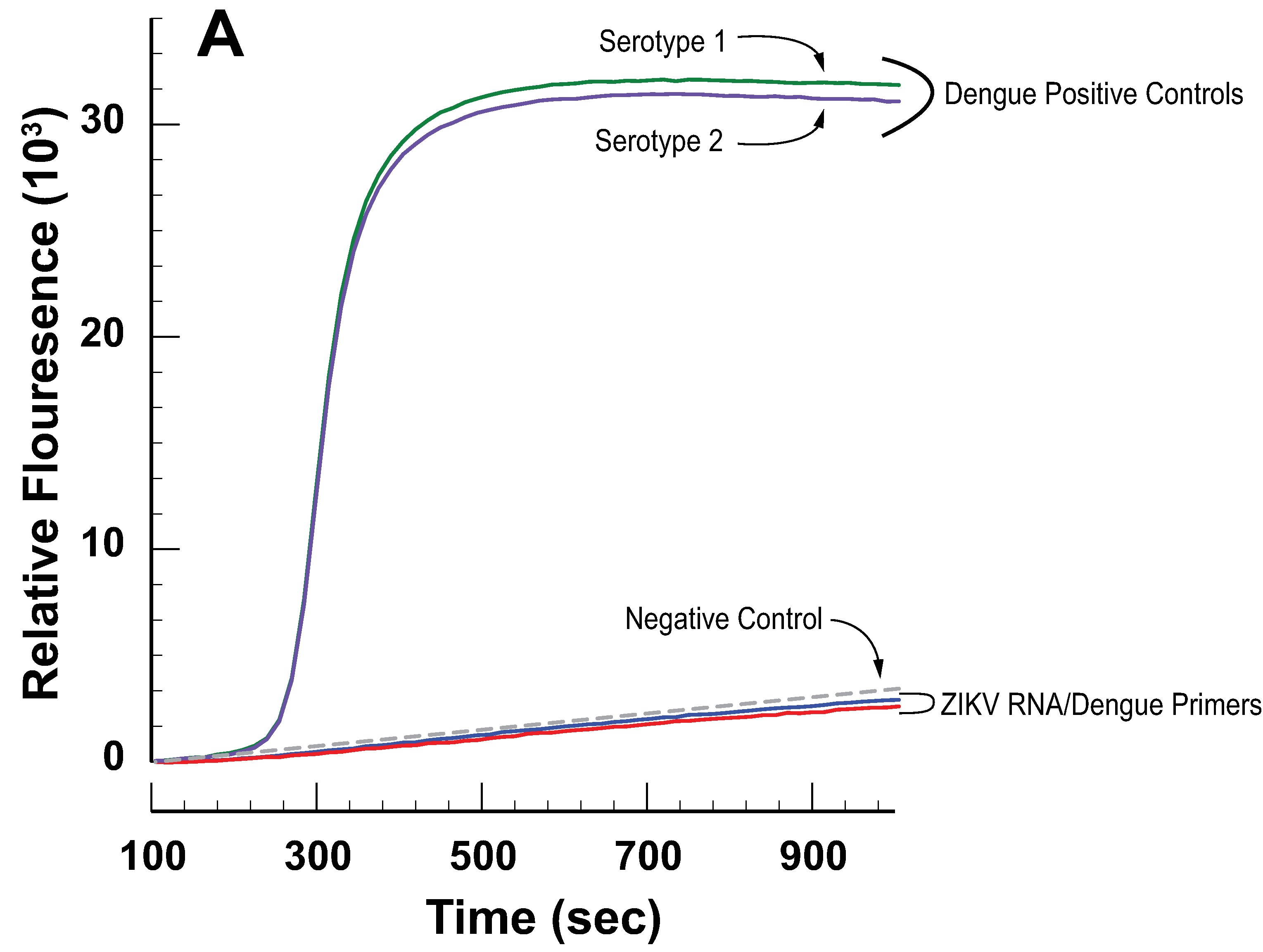

Supplement: S2 Fig — Graph of the amplification time (seconds) as a function of the fluorescence signal monitored with the Genie III device. No amplification detected in RT-LAMP assay using DENGV serotype 1 (blue) and 2 (red) genomic purified RNA as templates. DENGV serotype 1 (green) and 2 (purple) genomic purified RNA was used as a template for positive control. Distilled water instead of RNA was used as a negative control in the RT-LAMP assay. (TIF) [file pone.0192398.s002.tif]

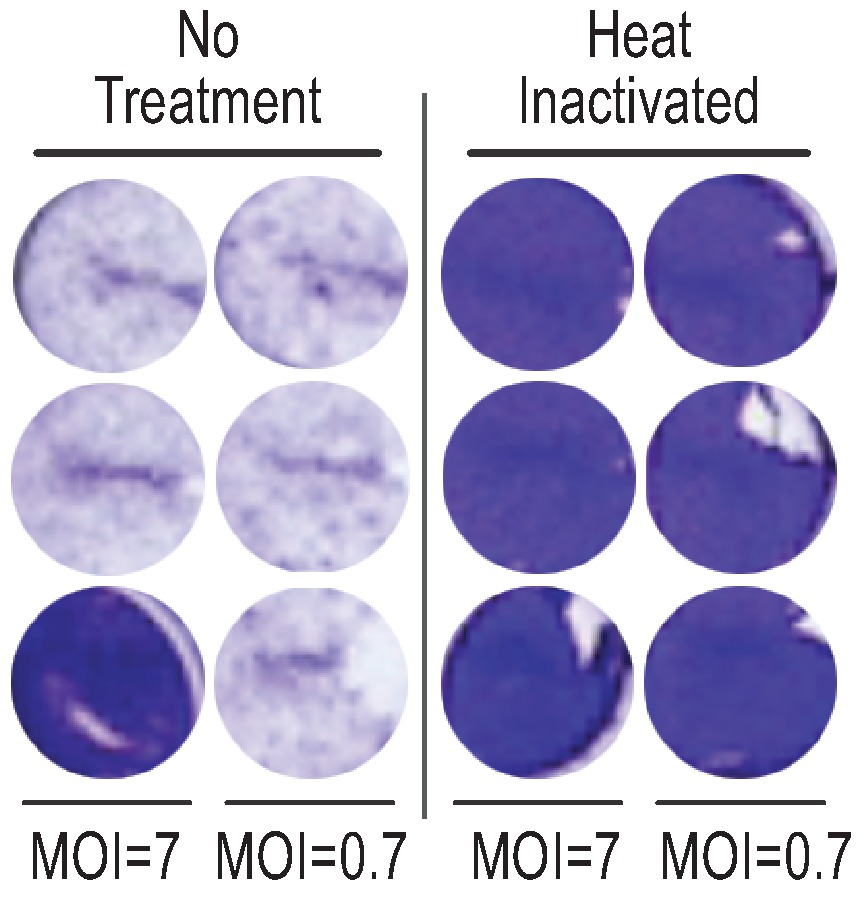

Supplement: S3 Fig — Infections at high and low MOIs with heat inactivated ZIKV at 90°C for 5 min did not show any detectable plaques. No heat inactivated ZIKV (no treatment) was used as a control for infected Vero cells. 2 replicates were used for high MOI infections and 3 for low MOI infection. The bottom well in the MOI 7 columns corresponds to Vero cells without virus used as an uninfected control. (TIF) [file pone.0192398.s003.tif]
