# Supplementary material for: Detection of Zika virus using reverse-transcription LAMP coupled with reverse dot blot analysis in saliva
Source: PLoS One. 2018 Feb 5;13(2):e0192398. doi: 10.1371/journal.pone.0192398 (PMC5798782; doi:10.1371/journal.pone.0192398)
Supplement: S1 Table — (DOCX) [file pone.0192398.s004.docx]

**S1 Table. Plaque assay to test heat inactivated ZIKV.**

| **ZIKV (PRVABC59)** | **Viral titer (pfu/ml)** | **MOI** | **Replicates** | **Infected Vero cells** |
| --- | --- | --- | --- | --- |
| ZIKV (No Treatment) | 7.6 x 10^6^ | 7.0 | 3 | 100% |
|  | 7.6 x 10^5^ | 0.7 | 4 | 100% |
| Heat-lysed ZIKV  (90˚C for 5 min) | 7.6 x 10^6^ | 7.0 | 3 | ND |
|  | 7.6 x 10^5^ | 0.7 | 4 | ND |

*pfu: plaque-forming units; MOI: multiplicity of infection
