# Supplementary material for: Detection of Zika virus using reverse-transcription LAMP coupled with reverse dot blot analysis in saliva
Source: PLoS One. 2018 Feb 5;13(2):e0192398. doi: 10.1371/journal.pone.0192398 (PMC5798782; doi:10.1371/journal.pone.0192398)
Supplement: S2 Table — (DOCX) [file pone.0192398.s005.docx]

**S2 Table. Microarray key for RDB assay.**

| **SC (0.25 μM)** | **SC (0.25 μM)** | **SC (0.25 μM)** |
| --- | --- | --- |
|  |  |  |
| ZIKV-Probe 1 (20 µM) | ZIKV-Probe 1 (2 µM) | ZIKV-Probe 1 (0.2 µM) |
|  |  |  |
| ZIKV-Probe 1 (20 µM) | ZIKV-Probe 2 (2 µM) | ZIKV-Probe 2 (0.2 µM) |
|  |  |  |
| ZIKV-Probe 3 (20 µM) | ZIKV-Probe 3 (2 µM) | ZIKV-Probe 3 (0.2 µM) |
|  |  |  |
| ZIKV-Probe 4 (20 µM) | ZIKV-Probe 4 (2 µM) | ZIKV-Probe 4 (0.2 µM) |
|  |  | **SC (0.25 μM)** |

SC: spotting control
