# Supplementary material for: Detection of Zika virus using reverse-transcription LAMP coupled with reverse dot blot analysis in saliva
Source: PLoS One. 2018 Feb 5;13(2):e0192398. doi: 10.1371/journal.pone.0192398 (PMC5798782; doi:10.1371/journal.pone.0192398)
Supplement: S3 Table — (DOCX) [file pone.0192398.s006.docx]

**S3 Table. Microarray key for RDB assay using Probe 4.**

| **SC (0.25 µM)** |  |  | **SC (0.25 µM)** |
| --- | --- | --- | --- |
| 20.0 µM | 20.0 µM | 0.625 µM | 0.625 µM |
| 10.0 µM | 10.0 µM | 0.32 µM | 0.32 µM |
| 5.0 µM | 5.0 µM | 0.156 µM | 0.156 µM |
| 2.5 µM | 2.5 µM | 0.078 µM | 0.078 µM |
| 1.25 µM | 1.25 µM | 0.039 µM | 0.039 µM |
|  |  |  |  |
|  |  |  |  |
|  |  |  |  |
| **Probe-2 (20 µM)** | **Probe-2 (2 µM)** | **Probe-2 (0.2 µM)** | **SC (0.25 µM)** |

SC: spotting control
